# Beyond interferon gamma - decreased cellular response to COVID-19 vaccination booster in patients with autoimmune inflammatory rheumatic diseases

**DOI:** 10.3389/fimmu.2025.1568439

**Published:** 2025-03-28

**Authors:** Jakub Wroński, Magdalena Massalska, Bożena Jaszczyk, Anna Felis-Giemza, Anna Kornatka, Magdalena Plebańczyk, Tomasz Burakowski, Brygida Kwiatkowska, Ewa Kuca-Warnawin, Marzena Ciechomska

**Affiliations:** ^1^ Department of Rheumatology, National Institute of Geriatrics, Rheumatology and Rehabilitation, Warsaw, Poland; ^2^ Department of Pathophysiology and Immunology, National Institute of Geriatrics, Rheumatology and Rehabilitation, Warsaw, Poland; ^3^ Department of Outpatient Clinics, National Institute of Geriatrics, Rheumatology and Rehabilitation, Warsaw, Poland; ^4^ Biologic Therapy Center, National Institute of Geriatrics, Rheumatology and Rehabilitation, Warsaw, Poland; ^5^ Department of Early Arthritis, National Institute of Geriatrics, Rheumatology and Rehabilitation, Warsaw, Poland

**Keywords:** COVID-19, autoimmune inflammatory rheumatic diseases, cellular immune response, vaccination, immunomodulatory drugs

## Abstract

The global COVID-19 pandemic has led to significant advancements in vaccine research, particularly regarding patients with autoimmune inflammatory rheumatic diseases (AIIRD). However, most studies have assessed the post-vaccination cellular response only by measuring the production of interferon-gamma. This study aimed to explore the post-vaccination cellular immune response in patients with AIIRD, with a focus on the effects of immunomodulatory drugs on different proteins involved in the cellular response and cytotoxicity. We analyzed blood samples from 54 patients - 16 healthy controls (HC) and 38 AIIRD patients - at three time points: before (T0), 4 weeks after (T1), and more than 6 months after (T2) a COVID-19 booster vaccination. Gene expression and concentration levels of 13 proteins involved in cellular immunity were assessed. Our study showed significantly reduced production of TNF at T0, IL-2 at T0 and T2, and perforin at T2 in AIIRD patients compared to HC. In AIIRD patients the expression of genes involved in cytotoxicity, including NRF2, TRAIL, cathepsin B, and cathepsin H was impaired. Both protein concentrations and gene expression were particularly altered in those treated with glucocorticoids, methotrexate, and biologic/targeted synthetic disease-modifying antirheumatic drugs (b/tsDMARDs). Among b/tsDMARDs only IL-17 inhibitors did not affect the cellular response. These findings suggest that COVID-19 vaccination elicits a weakened cellular response in patients with AIIRD, particularly those on immunosuppressive therapies, potentially compromising vaccine efficacy. Further studies are required to determine the clinical impact of these findings on long-term vaccine effectiveness in this population.

## 1 Introduction

The widespread use of vaccines and the natural evolution of the virus towards milder variants have ended the global COVID-19 pandemic. This period has yielded significant advancements in our understanding of vaccination strategies. Notably, patients with autoimmune inflammatory rheumatic diseases (AIIRD) have particularly benefited from the intensified and more comprehensive research efforts spurred by the pandemic, leading to improved insights into vaccine efficacy and safety within this population. Patients with AIIRD exhibit increased susceptibility to infections, a vulnerability arising both from the underlying pathology of the disease and the immunomodulatory therapies commonly employed in treatment. Consequently, the findings from recent research provide valuable insights. Among the key advancements is the emerging body of knowledge on the post-vaccination cellular immune response in this patient population.

Numerous studies have shown that patients with AIIRD achieve a cellular response after vaccination against COVID-19 (1–24), but in most studies lower than in healthy people (2, 4, 5, 7, 9–11, 13, 14, 18, 21–23, 25). Studies have shown a reduction in cellular response after specific immunomodulatory drugs - glucocorticoids (GCs) (2, 7–9, 26), conventional disease-modifying drugs (cDMARDs) such as methotrexate (MTX) (4, 6, 9, 13, 14, 16, 22, 23), mycophenolate mofetil (6, 25, 26), sulfasalazine (SSZ) (9), as well as biological and targeted synthetic disease-modifying drugs (b/tsDMARDs) such as JAK inhibitors (9, 25), TNF inhibitors (9, 23), IL-6 inhibitors (9), CD20 inhibitors (18), cytotoxic T lymphocyte-associated antigen 4 immunoglobulin fusion protein (10, 21), IL-17 inhibitors (23), and IL-12/23 inhibitors (22, 23). To date, most studies have assessed the cellular response only by measuring the production of interferon gamma (1–3, 6–11, 18, 26). However, multiple different cytokines, cytotoxic proteins, and transcription factors contribute to the anti-viral cellular response, the role of which in the post-vaccination response has been poorly understood.

In our previous studies on the booster dose of COVID-19 vaccination BNT162b2 in AIIRD patients, we showed that immunomodulatory drugs affect the cellular response more than the humoral one (9, 27). For this reason, we decided to look more closely at the post-vaccination cellular response. In blood samples collected from the previously described cohort of patients, we decided to determine the concentrations and gene expression of 13 different proteins involved in the cellular response and cytotoxicity. We selected proteins involved in the immune response (IL-2, TNF), apoptotic process/cell death (Fas, FasL, TNF, TRAIL, LT-α, and cathepsins), and leukocyte-mediated cytotoxicity (perforin, granzymes).

## 2 Methods

### 2.1 Patients

The study of selected proteins involved in the cellular response was performed on cryobanked biological material from patients recruited to a previously conducted study on the kinetics of the post-vaccination response after a booster dose of vaccination against COVID-19, BNT162b2, in patients with AIIRD (9). Blood samples were collected at the following time points: before the booster COVID-19 vaccination (T0), 4 weeks after the booster vaccination (T1), and after more than 6 months from the booster dose (T2). Patient characteristics (including the use of immunomodulatory drugs during the primary vaccination schedule and before the booster vaccination) were collected by qualifying physicians using a structured interview. Data regarding primary COVID-19 vaccinations and COVID-19 infections (both before and after booster vaccination) were gathered from both interviews and the national COVID-19 registry. Additionally, to detect asymptomatic COVID-19 infections, antibodies against SARS-CoV-2 nucleocapsid N were measured with a SARS-CoV N ELISA Kit (TestLine Clinical Diagnostics, Brno, The Czech Republic). Data regarding patient characteristics were blinded to the laboratory staff. The study protocol was approved by the hospital bioethics committee (KBT-3/2/2021). All participants signed informed consent for participation in the study. The study was conducted according to the Declaration of Helsinki.

### 2.2 Assessment of cellular response against viral antigens

A detailed analysis of the cellular response was performed on 54 patients - 16 patients from the healthy control (HC) group and 38 patients from the study (AIIRD) group at T0 and T1. Due to the withdrawal of some patients from the study group, the analysis at T2 included 45 patients - 16 patients from the HC group and 29 patients from the AIIRD group. For gene expression analysis and Enzyme-Linked Immunosorbent Assays (ELISAs), we used cryobanked peripheral blood mononuclear cells (PBMCs) and blood plasma samples previously prepared with the Quan-T-Cell SARS-CoV-2 test (Euroimmun, Lübeck, Germany). In the initial phase, freshly collected heparinized whole blood was incubated for 22 to 24 hours with the SARS-CoV-2 S1 antigen, which was coated at the base of a test tube. Additionally, the blood was incubated in a second tube as a negative control to evaluate non-specific background responses, and in a third tube serving as a positive control upon mitogen stimulation. After an incubation period, plasma and PBMCs were collected for further analysis. PBMCs were isolated from blood (only from tubes stimulated with viral antigens) by density gradient centrifugation with Ficoll-Paque (GE Healthcare, Uppsala, Sweden).

### 2.3 Enzyme-Linked Immunosorbent Assays (ELISAs)

ELISA assays were carried out to quantify the plasma concentrations of the following proteins associated with the cellular response: TNF, IL-2, perforin, and granzyme B. Details of the tests performed are shown in **Supplementary Table 1**. The results in the figures are shown as the ratio of the result from the sample stimulated with a viral antigen or mitogen to the result from the control sample (fold change).

### 2.4 Gene expression analysis

PBMCs were lysed in a Lysis Buffer RA1, and total RNA was isolated using the NucleoSpin RNA Mini kit (Macherey-Nagel, Duren, Germany). The concentration and purity of the isolated RNA were analyzed using a spectrophotometric reader (MultiSkan Go, Thermo Fisher Scientific, Waltham, MA, USA). A total of 40 ng of RNA was used for the reverse transcription reaction, which was performed using the High-Capacity cDNA Reverse Transcription Kit (Thermo Fisher Scientific, Waltham, MA, USA). The 10 µL PCR reaction included 2 µL RT product, 5 µL TaqMan Universal Master Mix, 0.5 µL probe mix of the TaqMan, and 2.5 µL of water (Genoplast, Rokocin, Poland). Reactions were performed at 50°C for 2 min, 95°C for 10 min, followed by 50 cycles at 95°C for 15 s and 60°C for 1 min. Samples were analyzed in triplicate using the QuantStudio 5 qRT-PCR machines (Thermo Fisher Scientific, Waltham, MA, USA). Gene expression was evaluated using ΔΔCT-method. The list of tested genes is presented in **Supplementary Table 2**.

### 2.5 Statistics

The compliance of the data with the normal distribution was assessed using the Shapiro–Wilk test. The significance of the observed differences between the two groups was assessed using the Student’s T test for variables with a normal distribution, the Mann–Whitney U test for variables without a normal distribution, and categorical variables the Fisher’s exact test. The significance of the results after adjusting for confounding factors (listed in **Table 1**) was checked by linear regression. Statistical analysis was performed using Statistica 13.3 software (StatSoft Polska, Cracow, Poland) and figures were created using GraphPad Prism software version 7 (GraphPad Software, Boston, MA, USA).

**Table 1:** Patient characteristics.

	Inflammatory arthritis (n=38)	Healthy control (n=16)	difference
Age (mean ± SD)	52.7 ± 13.7	41.8 ± 9.0	p=0.005
Sex – female, n (%)	24 (63.2%)	10 (62.5%)	ns
BMI (mean ± SD)	27.7 ± 5.3	27.7 ± 5.6	ns
Smoking, n (%) - current - past	4 (10.5%) 11 (28.9%)	1 (6.3%) 1 (6.3%)	ns ns
Vaccine booster dose, n (%) - BNT162b2	38 (100%)	16 (100%)	ns
Heterologous Booster Vaccine, n (%) - ChAdOx1-S - mRNA-1273 - JNJ-78436735	9 (23.7%) 4 (10.5%) 3 (8%) 2 (5.2%)	3 (18,8%) 2 (12.5%) 1 (6.3%) -	ns
Days after booster vaccination (median, min-max) - first point - second point	31 (22–52) 205.6 (167–290)	31 (27–77) 199 (175–234)	ns ns
COVID-19 infection after booster vaccination, n (%)	5 (13.2%)	3 (18.8%)	ns

n, number; ns, nonsignificant.

### 2.6 Functional and pathway analysis

The genes CTSH, CTSB, CTSC, GZMB, GZMA, TNFSF10/TRAIL, LTA, NFE2L2/NRF2, FASLG/FASL, FAS, IL2, PRF1, TNF, which form protein-protein interaction (PPI) networks that fulfill biological processes, were analyzed. The construction of the PPI networks and Gene Ontology enrichment were studied using the Search Tool for the Retrieval of Interacting Genes (STRING, v12.0) (https://www.string-db.org/(accessed on 7 January 2025)). Confidence scores > 0.8 were set as significant. STRING is a database that complies known or predicted protein interactions derived from high-throughput experiments, genomic analysis, conservative co-expression, and previously known literature (28).

## 3 Results

Patient characteristics are presented in **Table 1**. There were no significant differences between the groups, apart from the older age of the AIIRD group (p=0.005). Therefore all subsequent analyses were age-adjusted. In both groups, a similar percentage of subjects had COVID-19 after the booster dose, based on the presence of antibodies against SARS-CoV-2 nucleocapsid.

**Figure 1:**
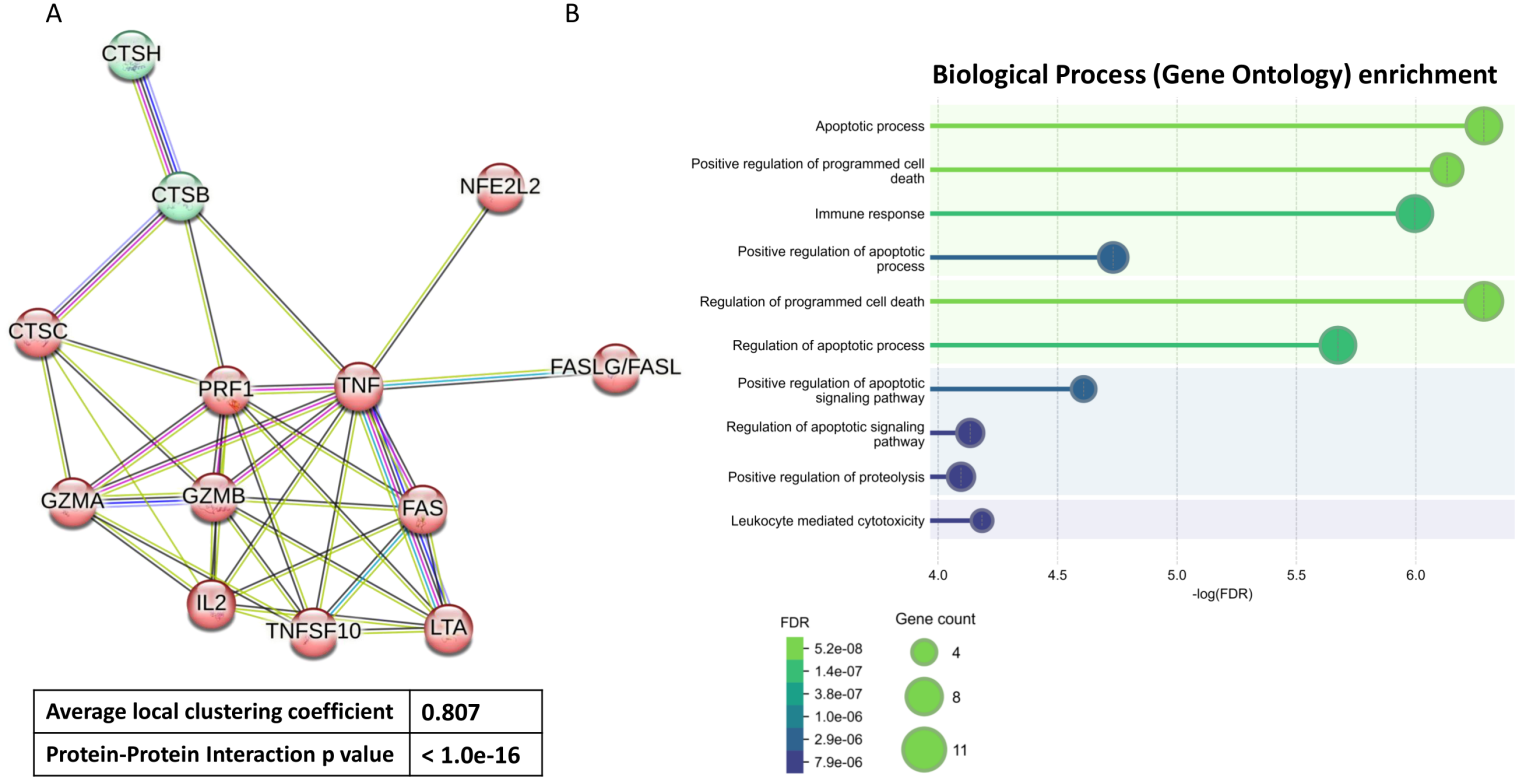
Functional enrichment analysis. Protein-protein Interaction based on String analysis **(A)** and different Biological Process based on Gene Ontology enrichment analysis **(B)** of 13 selected proteins. These proteins coding genes include *CTSH, CTSB, CTSC, GZMB, GZMA, TNFSF10/TRAIL, LTA, NFE2L2/NRF2, FASLG/FASL, FAS, IL2, PRF1, TNF*. FDR (False Discovery Rate).

To find the functional effects of 13 selected proteins we used the STRING database. Indeed, the network, restricted to MCL stochastic flow clustering, revealed robust interactions between these 13 proteins within highly interconnected groups (**Figure 1A**). These strong interactions were quantified by a Protein-Protein Interaction (PPI) enrichment p-value reaching <1.0e-16 and an average local clustering coefficient reaching 0.807. The strength of these interactions was further supported by Gene Ontology (GO) enrichment analysis, which identified 10 genes involved in immune response (**Figure 1B**). Additionally, between 5 to 10 genes were linked to apoptosis and other regulated forms of programmed cell death, 4 genes were associated with leukocyte-mediated cytotoxicity, and 6 genes participated in positive regulation of proteolysis.

**Figure 2:**
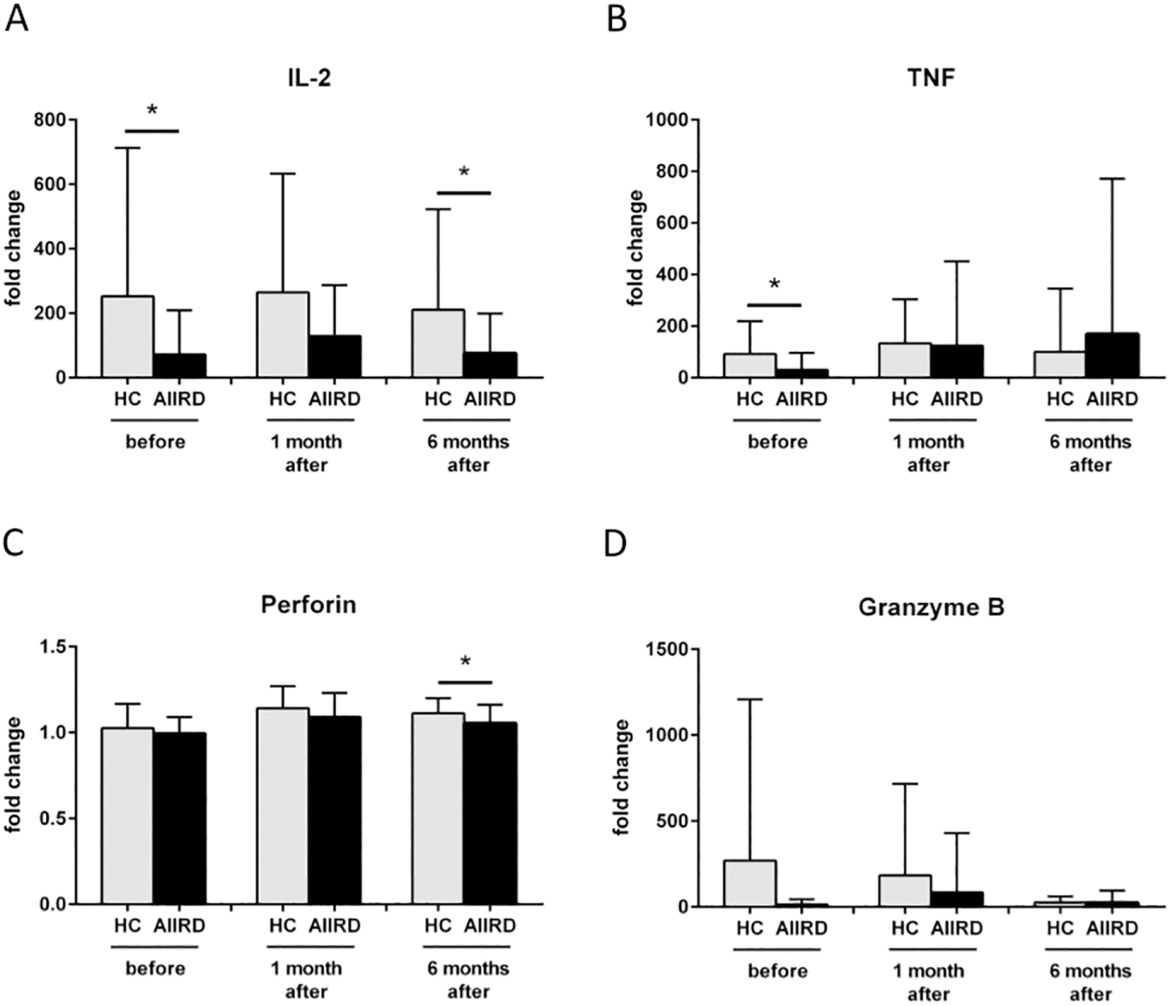
Fold change in protein secretion of IL-2 **(A)**, TNF **(B)**, Perforin **(C)**, Granzyme B **(D)** involved in cellular response before, 1 month and 6 months following COVID vaccination in HC and AIIRD patients’ plasma upon full blood simulation with SARS-CoV-2 antigen. AIIRD, autoimmune inflammatory rheumatic diseases; HC, healthy controls. *P<0.05.

We then investigated the differences in the concentration of the cellular response and cytotoxicity proteins between patients with AIIRD and the control group after viral protein stimulation (**Figure 2**). Decreased production of IL-2 at T0 (p=0.03) and T2 (p=0.03), TNF at T0 (p=0.02), and perforin at T2 (p=0.02) were observed in patients from the study group compared to the control group. No difference in granzyme B production between groups was detected. Also, no significant differences after mitogen stimulation in the production of cytotoxic molecules were observed (data not shown).

**Figure 3:**
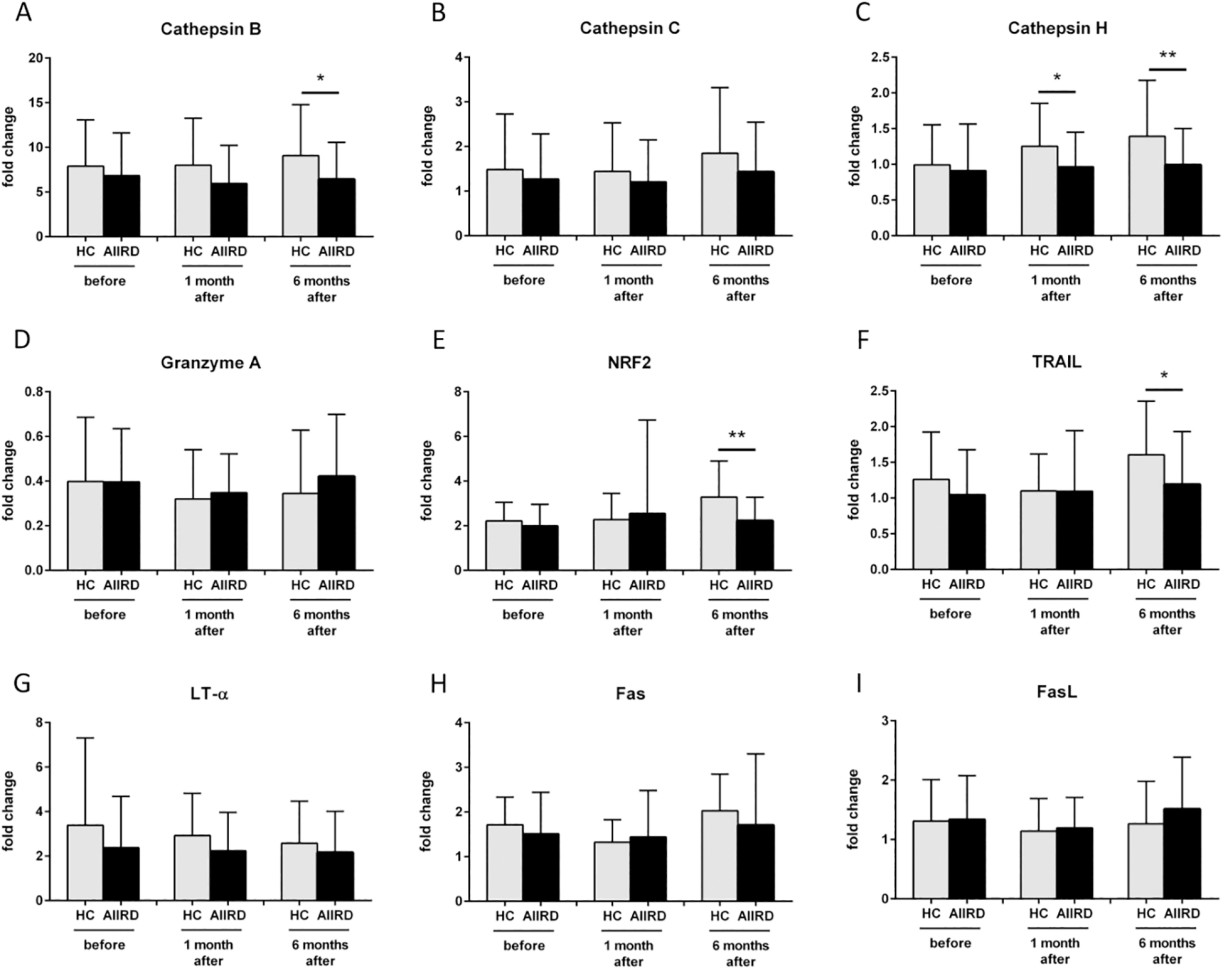
Gene expression of Cathepsin B **(A)**, Cathepsin C **(B),** Cathepsin H **(C)**, Granzyme A **(D)**, NRF2 **(E)**, TRAIL **(F)**, LT-α **(G)**, Fas **(H)**, FasL **(I)** involved in cellular response before, 1 month and 6 months following COVID vaccination in HC and AIIRD patients’ PBMC upon full blood simulation with SARS-CoV-2 antigen. AIIRD, autoimmune inflammatory rheumatic diseases; HC, healthy controls; *P<0.05 **P<0.01.

The comparison of gene expression between both groups is shown in **Figure 3**. Decreased expression of cathepsin B CTSB (p=0.049), NRF2 (p=0.004), and TNFSF10/TRAIL (p=0.02) genes at T2, as well as decreased cathepsin H CTSH gene at T1 (p=0.02) and T2 (p=0.004), were observed in AIIRD patients compared to the HC. In contrast, cathepsin C CTSC, granzyme A GZMA, LT-α LTA, FAS, and FASLG/FASL were not significantly different between groups.

**Figure 4:**
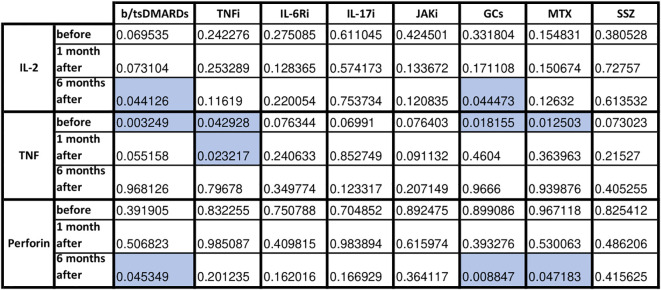
Statistical significance in fold change in cytotoxicity-related proteins before, 1 month and 6 months following COVID vaccination in HC and AIIRD patients’ plasma upon full blood simulation with SARS-CoV-2 antigen. Blue color – statistically significant decrease compared to healthy controls (HC).

Next, we examined the effect of each immunomodulatory drug on the production of cellular response and cytotoxicity proteins after viral protein stimulation (**Figure 4**). The greatest impact on the components of the post-vaccination cellular response was observed with biological drugs and GCs (decreased levels of IL-2, TNF, and perforin), with some effect of MTX (decreased levels of TNF and perforin). There was also a significant decrease in the production of granzyme (T0 p=0.008) and TNF (T2 p=0.04) in patients using b/tsDMARDs in combination with cDMARDs vs. monotherapy (data not shown).

**Figure 5:**
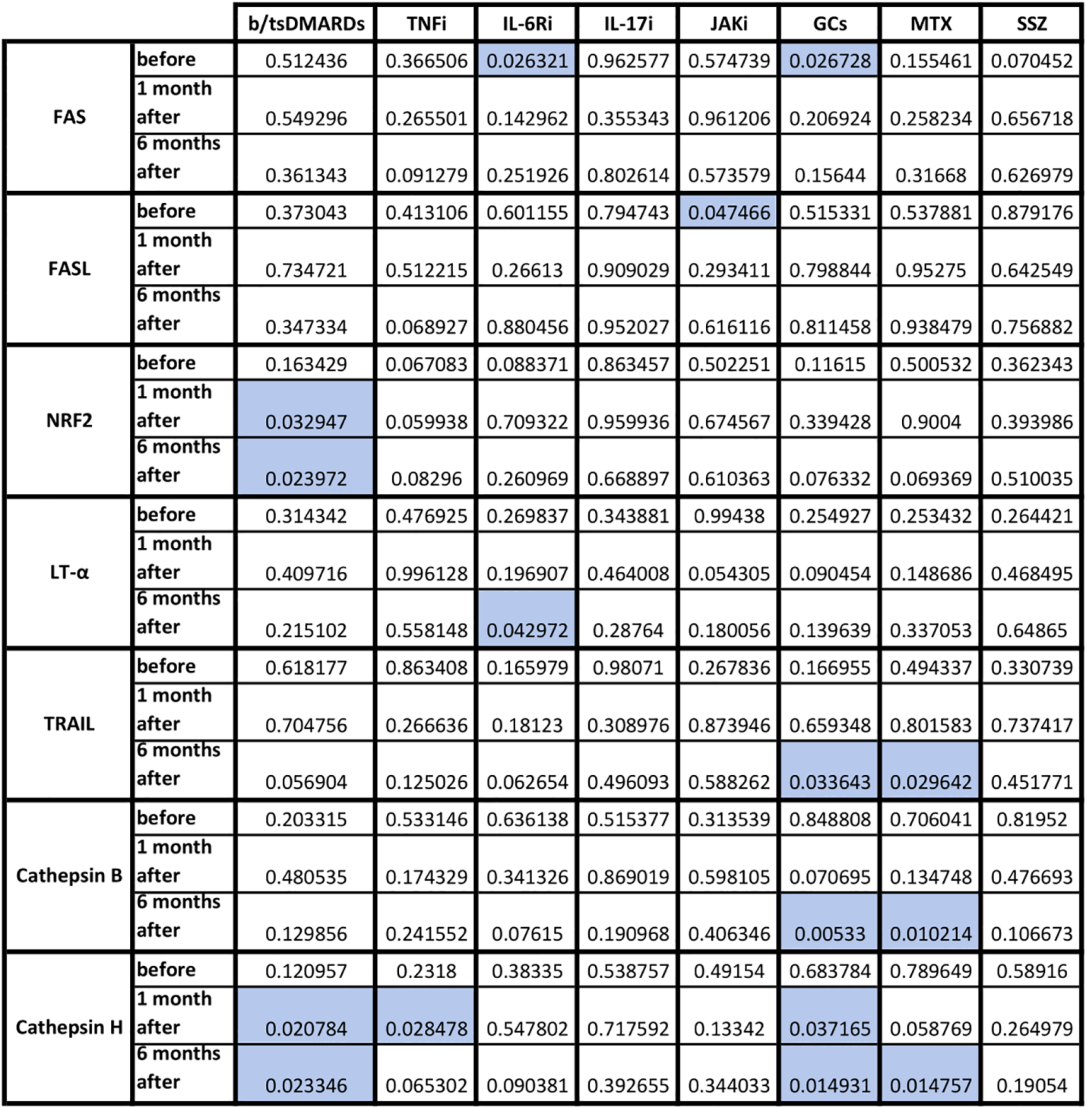
Statistical significance in fold change in cytotoxicity-related genes following COVID vaccination in HC and AIIRD patients’ PBMC upon full blood simulation with SARS-CoV-2 antigen. Blue color – statistically significant decrease compared to healthy controls (HC).

The effect of each immunomodulatory drug on the difference in cellular response and cytotoxicity gene expression of both groups is shown in **Figure 5**. After b/tsDMARDs decreased levels of NRF2 and cathepsin H CTSH gene expression were observed. GCs affected FAS, TNFSF10/TRAIL, cathepsin B CTSB, and cathepsin H CTSH expression, with a similar pattern (excluding FAS) observed after MTX. Among biological and synthetic targeted drugs, only IL-17 inhibitors did not affect any cytotoxic gene expression. Patients who combined DMARDs with GCs had lower LT-α LTA gene expression at T0 (p=0.04) than patients treated only with DMARDs (data not shown).

## 4 Discussion

Our study demonstrated a reduced immunogenic response to COVID-19 vaccination in patients with AIIRD. This was characterized by limited production and expression of genes involved in various components of the cellular immune response and cytotoxicity. Although we identified robust interactions between 13 proteins involved in cellular cytotoxicity, apoptosis, and immune regulation through String analysis (**Figure 1**), only a selected subset of these molecules was affected by immunosuppressive therapy in AIIRD patients compared to HC. The comprehensive summary of our results with comparison to the literature data is presented in **Table 2**.

**Table 2:** Summary of the impact of COVID-19 vaccination on selected molecules related to cellular immunity.

	After COVID-19 vaccination	Previous studies in AIIRD patients – difference compared to HC	Our study in AIIRD patients – difference compared to HC
IL-2	↑ 5, 17, 19–23, 30	ns^17^, ^19^, ^20^/↓^5^, ^21^–^23^, ^25^	↓
TNF	ns 22, 30, 34, 35/↑ 17, 19, 21, 24 ,32, 33	ns^12^, ^13^, ^17^, ^19^, ^24^, ^25^/↓^21^	↓
Perforin	↑^22^, ^23^, ^30^, ^32^, ^37^–^39^	ns^22^/↓^23^	↓
Granzymes	↑^4^, ^5^ ,^22^, ^23^, ^30^, ^32^, ^35^, ^37^–^39^	ns^22^/↓^4^, ^5^, ^23^	ns
FasL	↑^22^, ^23^	ns^22^/↓^23^	ns
Fas	↑^39^	not studied	ns
NRF2	↑^43^	not studied	↓
TRAIL	ns	not studied	↓
LT α	↑^48^	not studied	ns
Cathepsin B	not studied	not studied	↓
Cathepsin H	not studied	not studied	↓
Cathepsin C	not studied	not studied	ns

ns, nonsignificant difference.

↑ upregulated, ↓ downregulated.

Among others, we investigated the concentration of IL-2 and TNF – cytokines which are some of the best-studied components of the cellular response after vaccination against COVID-19. Our experiments showed that basic cellular response containing IL-2 and TNF production was induced by vaccination, but was lower in AIIRD patients.

IL-2 polarizes the immune response towards Th1, is a growth factor for cytotoxic lymphocytes and NK, and affects Treg (29). After COVID-19 vaccination, IL-2 levels increase (5, 17, 19–23, 30). In AIIRD patients IL-2 levels also increase after vaccination, but most studies shown lower levels of IL-2 in patients with AIIRD compared to HC (5, 21–23, 25). The results of our study support this observation (**Figure 2A**), with lower IL-2 levels seen in patients treated with bDMARDs and GCs (**Figure 4**).

The role of TNF in the antiviral response includes inducing apoptosis of infected cells, modulating innate immune responses, and promoting the infiltration of macrophages, dendritic cells, NK cells, and neutrophils to the affected area (31). In the case of post-vaccination response, the studies on TNF are not conclusive, as a number of studies showed increased TNF response after vaccination (17, 19, 21, 24, 32, 33), although some didn’t (22, 30, 34, 35). Most studies did not show any differences in TNF levels/number of TNF-producing cells between HC and AIIRD (12, 13, 17, 19, 24, 25). Contrary, in our study, similarly to results presented by Farroni et al. (21), decreased level of TNF production was seen in AIIRD patients (**Figure 2B**), which may impact antiviral response, particularly in patients treated with b/tsDMARDs, TNFi, GCs, and MTX (**Figure 4**).

Several cytotoxicity mechanisms can be observed in post-vaccination response – among them are those connected with cytoplasmic lytic grains (perforins, granzymes, cathepsins), oxidative stress, or those inducing apoptosis and connected with receptors for TNF molecules (Fas/FasL, TRAIL and LT-α). In our study, the potential of lytic grain production and protection against oxidative stress was lower in AIIRD patients as compared to HC.

One of the best-studied cytotoxicity protein in the post-vaccination response is perforin. Perforin plays a key role in the destruction of virally infected host cells, generating pores in the target cell membrane allowing entry of effector molecules (such as granzymes and granulysin) and subsequent cell death. Reduced perforin and dysregulated NK function were observed in patients with severe forms of COVID-19 (36). Perforin concentration increases after COVID-19 vaccination (22, 23, 30, 32, 37–39). In AIIRD patients, perforin levels after vaccination did not differ from HC (22, 23), though in TNFi-treated patients decreased faster between vaccination doses than in HC (23). This may be due to the subtlety of the effect of vaccination on perforin production, as, for example, a study on patients treated with RTX and HC did not show any perforin response following vaccination (19). In our study perforin levels were decreased compared to HC after six months from the vaccination (**Figure 2C**), with the greatest effect in patients treated with b/tsDMARDs, GCs, and MTX (**Figure 4**).

The other key cytotoxicity proteins that play a role in post-vaccination response are granzymes A and B. Granzymes are proteases released by cytoplasmic granules within cytotoxic T cells and NK cells, inducing target-cell lysis and apoptosis in the infected viruses’ cells. COVID-19 vaccination increases granzymes concentration/number of granzyme-producing cells – granzyme A (30), B (4, 35, 38) or both (5, 22, 23, 32, 37, 39). Similarly to perforin difference in concentration of granzymes after COVID-19 vaccination in AIIRD and HC may be slight – one study showed a similar increase of granzymes A and B (22), while others indicated lower post-vaccine production in AIIRD (4, 5, 23). In our study granzyme B concentration and granzyme A GZMA gene expression were not statistically different between patients and HC groups (**Figures 2D**, **3D** respectively).

Other proteases, such as cathepsins, play a different role in viral infections. On the one hand, they can be used by viruses to enter the cell (an increased level of cathepsin B promotes entry, including SARS-CoV-2 infection), on the other hand, they help fight viruses - they are associated with the presentation of antigens (a higher level of cathepsin H increases presentation, as also showed in COVID-19) or help limit viral replication (increased level of cathepsin C) (40, 41). In our study, patients with AIIRD showed lower expression of both cathepsin B CTSB (**Figure 3A**) and H CTSH (**Figure 3C**) genes, especially among patients treated with biologics (cathepsin H), GCs, and MTX (both B and H) (**Figure 5**). The significance of the observed associations on the post-vaccination response remains unknown.

Another molecule that we studied was NRF2 which is one of the key transcription factors in the human body that protects cells against oxidative stress by inducing the expression of multiple genes involved in immunity and inflammation, including those with antiviral action (42). NRF2, among others, regulates innate immune response, and cytosolic DNA sensing, and inhibits the replication of viruses through a type I IFN-independent pathway (43). Only one study assessed NRF2 expression following COVID-19 vaccination and showed increased NRF2 expression following vaccination (43). In our study decreased NRF2 gene expression was detected in AIIRD patients compared to HC (**Figure 3E**), especially in b/tsDMARDs treated patients (**Figure 5**).

Induction of apoptosis in virus-infected cells by cytotoxic T lymphocytes and NK cells may be based not only on granule exocytosis but also on other pathways using molecules for TNF receptors, like TRAIL, LT-α and Fas/FasL pathway (44). Our study showed a slightly lower apoptosis pathway gene expression after some immunomodulatory drugs.

TRAIL can induce apoptosis in virally infected cells, and regulate cytokine production, but also is responsible for the clinical course of some viral infections and can be used by viruses to increase viral replication (45). Only one study assessed TRAIL levels after COVID-19 vaccination and did not show a post-vaccination increase in TRAIL (46). In our study, among AIIRD patients, we noticed lower TNFSF10/TRAIL gene expression compared to HC (**Figure 3F**), especially in patients treated with GCs and MTX (**Figure 5**).

Another cytotoxic protein that we assessed in our study, was LT-α. The role of LT-α in viral infections is not directly related to cytotoxicity but rather results from the regulation of the immune system by controlling the development and maintenance of lymphoid organs, lymphoid organ integrity during viral infections, and activation of production of type I interferons (47). In patients vaccinated against COVID-19, higher LT-α levels have been shown to correlate with a better humoral response (48). In our study, LT-α LTA gene expression was found to be reduced only in patients using combined DMARDs with GCs therapy (data not shown).

The Fas (Fas receptor/CD95) is a death receptor on the surface of cells that leads to apoptosis if it binds its ligand, cytokine FasL (CD95L). The role of Fas/FasL in the post-vaccination response against COVID-19 is poorly understood, but it has been shown vaccination increases soluble FasL (22, 23) or both Fas and FasL production (39). Previous studies have shown a reduction in FasL both after TNFi (22) and in AIIRD in general (23). In our study, only GCs lowered FAS gene expression after vaccination compared to HC, while FASLG/FASL gene expression was lowered only by JAK inhibitors (**Figure 5**).

The strength of our study is the examination of multiple factors of the cellular response and cytotoxicity, which allows us to expand current knowledge of the post-vaccination response, not only in AIIRD patients but in general. These selected molecules are strongly interconnected as demonstrated by String analysis. Although most of the components of cellular response and cytotoxicity that we examined were studied after COVID-19 vaccination in the general population (NRF2, TRAIL, LT-α, Fas), only some were studied in people with AIIRD (IL-2, TNF, perforin, granzymes, FasL). Moreover, the expression of genes encoding cathepsins B and H is the first study of their level after COVID-19 vaccination in general. An obvious limitation of the study is the small group of patients, which most likely allowed us to detect only the strongest effect of AIIRD themselves and immunomodulatory drugs on the postvaccination cellular response. Moreover, the small number of patients and different group sizes at different time points do not allow for comparison of results over time since vaccination. An additional limitation of the study is that most of the components of the cellular response studied were measured only based on protein production or gene expression, not both at once.

## 5 Conclusions

Nevertheless, our study confirms the results of previous studies that although vaccination in AIIRD induces a cellular response, it is lower than in HC. The post-vaccination cellular response to COVID-19 vaccination in AIIRD patients is profoundly impaired on many different levels, going beyond the standardly assessed production of interferon-gamma. Our study also indicates groups of immunomodulatory drugs that limit the cellular response to a greater extent than others. However, the clinical significance of the observed results, such as how impaired cellular immunity affects vaccine effectiveness, remains unclear and requires further research.

## Data Availability

The raw data supporting the conclusions of this article will be made available by the authors, without undue reservation.

## References

[1] Hamad Saied M van Straalen JW de Roock S Verduyn Lunel FM de Wit J de Rond LGH. Humoral and cellular immunogenicity, effectiveness and safety of COVID-19 mRNA vaccination in patients with pediatric rheumatic diseases: A prospective cohort study. Vaccine. (2024) 42:1145–53. doi: 10.1016/J.VACCINE.2024.01.047 38262809

[2] Monti S Fornara C Delvino P Bartoletti A Bergami F Comolli G. Immunosuppressive treatments selectively affect the humoral and cellular response to SARS-CoV-2 in vaccinated patients with vasculitis. Rheumatol (Oxford). (2022) 62:keac365. doi: 10.1093/RHEUMATOLOGY/KEAC365 PMC9278207 35736379

[3] Prendecki M Clarke C Edwards H McIntyre S Mortimer P Gleeson S. Humoral and T-cell responses to SARS-CoV-2 vaccination in patients receiving immunosuppression. Ann Rheum Dis. (2021) 80:1322–9. doi: 10.1136/annrheumdis-2021-220626 PMC8350975 34362747

[4] Haberman RH Herati RS Simon D Samanovic M Blank RB Tuen M. Methotrexate hampers immunogenicity to BNT162b2 mRNA COVID-19 vaccine in immune-mediated inflammatory disease. Ann Rheum Dis. (2021) 80:1339. doi: 10.1136/ANNRHEUMDIS-2021-220597 34035003 PMC8219484

[5] Sieiro Santos C Calleja Antolin S Moriano Morales C Garcia Herrero J Diez Alvarez E Ramos Ortega F. Immune responses to mRNA vaccines against SARS-CoV-2 in patients with immune-mediated inflammatory rheumatic diseases. RMD Open. (2022) 8:e001898. doi: 10.1136/RMDOPEN-2021-001898 34987093 PMC9065768

[6] Moyon Q Sterlin D Miyara M Anna F Mathian A Lhote R. BNT162b2 vaccine-induced humoral and cellular responses against SARS-CoV-2 variants in systemic lupus erythematosus. Ann Rheum Dis. (2022) 81:575–83. doi: 10.1136/ANNRHEUMDIS-2021-221097 PMC8494536 34607791

[7] Renaudineau Y Sailler L Abravanel F Izopet J Delourme A Biotti D. Glucocorticoid use as a cause of non-cellular immune response to SARS-Cov2 Spike in patients with immune system diseases. J Autoimmun. (2022) 133:102912. doi: 10.1016/J.JAUT.2022.102912 36115213 PMC9464584

[8] Krasselt M Wagner U Nguyen P Pietsch C Boldt A Baerwald C. Humoral and cellular response to COVID-19 vaccination in patients with autoimmune inflammatory rheumatic diseases under real-life conditions. Rheumatol (Oxford). (2022) 61:SI180–8. doi: 10.1093/RHEUMATOLOGY/KEAC089 PMC8903382 35143648

[9] Wroński J Jaszczyk B Roszkowski L Felis-Giemza A Bonek K Kornatka A. The kinetics of humoral and cellular responses after the booster dose of COVID-19 vaccine in inflammatory arthritis patients. Viruses. (2023) 15:620. doi: 10.3390/V15030620 36992329 PMC10052973

[10] Farroni C Picchianti-Diamanti A Aiello A Nicastri E Laganà B Agrati C. Kinetics of the B- and T-cell immune responses after 6 months from SARS-coV-2 mRNA vaccination in patients with rheumatoid arthritis. Front Immunol. (2022) 13:846753. doi: 10.3389/FIMMU.2022.846753 35309297 PMC8924958

[11] Sidler D Born A Schietzel S Horn MP Aeberli D Amsler J. Trajectories of humoral and cellular immunity and responses to a third dose of mRNA vaccines against SARS-CoV-2 in patients with a history of anti-CD20 therapy. RMD Open. (2022) 8:e002166. doi: 10.1136/RMDOPEN-2021-002166 35361691 PMC8971359

[12] Furer V Eviatar T Zisman D Peleg H Paran D Levartovsky D. Immunogenicity and safety of the BNT162b2 mRNA COVID-19 vaccine in adult patients with autoimmune inflammatory rheumatic diseases and in the general population: a multicenter study. Ann Rheum Dis. (2021) 80:1330–8. doi: 10.1136/ANNRHEUMDIS-2021-220647 34127481

[13] Eviatar T Pappo A Freund T Friedlander Y Elkayam O Hagin D. Cellular immune response to the anti-SARS-CoV-2 BNT162b2 mRNA vaccine in pediatric autoimmune inflammatory rheumatic disease patients and controls. Clin Exp Immunol. (2024) 217:167–72. doi: 10.1093/CEI/UXAE044 PMC11239557 38767466

[14] Mahil SK Bechman K Raharja A Domingo-Vila C Baudry D Brown MA. Humoral and cellular immunogenicity to a second dose of COVID-19 vaccine BNT162b2 in people receiving methotrexate or targeted immunosuppression: a longitudinal cohort study. Lancet Rheumatol. (2022) 4:e42–52. doi: 10.1016/S2665-9913(21)00333-7 PMC8577228 34778846

[15] Smetanova J Strizova Z Sediva A Milota T Horvath R. Humoral and cellular immune responses to mRNA COVID-19 vaccines in patients with axial spondyloarthritis treated with adalimumab or secukinumab. Lancet Rheumatol. (2021) 4:e163. doi: 10.1016/S2665-9913(21)00393-3 34957418 PMC8691856

[16] Andreica I Blazquez-Navarro A Sokolar J Anft M Kiltz U Pfaender S. Different humoral but similar cellular responses of patients with autoimmune inflammatory rheumatic diseases under disease-modifying antirheumatic drugs after COVID-19 vaccination. RMD Open. (2022) 8:e002293. doi: 10.1136/RMDOPEN-2022-002293 36104115 PMC9475968

[17] Fabris M De Marchi G Domenis R Caponnetto F Guella S Dal Secco C. High T-cell response rate after COVID-19 vaccination in belimumab and rituximab recipients. J Autoimmun. (2022) 129:102827. doi: 10.1016/J.JAUT.2022.102827 35427999 PMC8995326

[18] Moor MB Mani L Sidler D Horn MP Iype JM. Humoral and cellular responses to mRNA vaccines against SARS-CoV-2 in patients with a history of CD20 B-cell-depleting therapy (RituxiVac): an investigator-initiated, single-center, open-label study. Lancet Rheumatol. (2021) 3:e789. doi: 10.1016/S2665-9913(21)00251-4 34514436 PMC8423431

[19] Bitoun S Henry J Desjardins D Vauloup-Fellous C Dib N Belkhir R. Rituximab impairs B cell response but not T cell response to COVID-19 vaccine in autoimmune diseases. Arthritis Rheumatol. (2022) 74:927–33. doi: 10.1002/ART.42058/ABSTRACT PMC9011892 34962357

[20] Mahil SK Bechman K Raharja A Domingo-Vila C Baudry D Brown MA. The effect of methotrexate and targeted immunosuppression on humoral and cellular immune responses to the COVID-19 vaccine BNT162b2: a cohort study. Lancet Rheumatol. (2021) 3:e627. doi: 10.1016/S2665-9913(21)00212-5 34258590 PMC8266273

[21] Farroni C Aiello A Picchianti-Diamanti A Laganà B Petruccioli E Agrati C. Booster dose of SARS-CoV-2 messenger RNA vaccines strengthens the specific immune response of patients with rheumatoid arthritis: A prospective multicenter longitudinal study. Int J Infect Dis. (2022) 125:195. doi: 10.1016/J.IJID.2022.10.035 36328289 PMC9622025

[22] Dayam RM Law JC Goetgebuer RL Chao GYC Abe KT Sutton M. Accelerated waning of immunity to SARS-CoV-2 mRNA vaccines in patients with immune-mediated inflammatory diseases. JCI Insight. (2022) 7(11):e159721. doi: 10.1172/JCI.INSIGHT.159721 35471956 PMC9220925

[23] Cheung MW Dayam RM Shapiro JR Law JC Chao GYC Pereira D. Third and fourth vaccine doses broaden and prolong immunity to SARS-coV-2 in adult patients with immune-mediated inflammatory diseases. J Immunol Author Choice. (2023) 211:351. doi: 10.4049/JIMMUNOL.2300190 PMC10352588 37326480

[24] Szebeni GJ Gémes N Honfi D Szabó E Neuperger P Balog J. Humoral and cellular immunogenicity and safety of five different SARS-coV-2 vaccines in patients with autoimmune rheumatic and musculoskeletal diseases in remission or with low disease activity and in healthy controls: A single center study. Front Immunol. (2022) 13:846248/FULL. doi: 10.3389/FIMMU.2022.846248/FULL 35432314 PMC9008200

[25] De Santis M Motta F Isailovic N Clementi M Criscuolo E Clementi N. Dose-dependent impairment of the immune response to the moderna-1273 mRNA vaccine by mycophenolate mofetil in patients with rheumatic and autoimmune liver diseases. Vaccines (Basel). (2022) 10:801. doi: 10.3390/VACCINES10050801 35632557 PMC9144166

[26] Yang LM Costales C Ramanathan M Bulterys PL Murugesan K Schroers-Martin J. Cellular and humoral immune response to SARS-CoV-2 vaccination and booster dose in immunosuppressed patients: An observational cohort study. J Clin Virol. (2022) 153:105217. doi: 10.1016/J.JCV.2022.105217 35714462 PMC9188451

[27] Wroński J Jaszczyk B Roszkowski L Felis-Giemza A Bonek K Kornatka A. Humoral and cellular immunogenicity of COVID-19 booster dose vaccination in inflammatory arthritis patients. Front Immunol. (2022) 13:1033804. doi: 10.3389/FIMMU.2022.1033804 36389719 PMC9659732

[28] Szklarczyk D Franceschini A Kuhn M Simonovic M Roth A Minguez P. The STRING database in 2011: functional interaction networks of proteins, globally integrated and scored. Nucleic Acids Res. (2011) 39:D561. doi: 10.1093/NAR/GKQ973 21045058 PMC3013807

[29] Su R Zhang T Wang H Yan G Wu R Zhang X. New sights of low dose IL-2: Restoration of immune homeostasis for viral infection. Immunology. (2024) 171:324–38. doi: 10.1111/IMM.13719 37985960

[30] Familiar-Macedo D Vieira Damasco P Fiestas Solórzano VE Carnevale Rodrigues J Sampaio de Lemos ER Barreto dos Santos F. Inflammatory and cytotoxic mediators in COVID-19 patients and in ChAdOx1 nCoV-19 (AZD1222) vaccine recipients. Cytokine. (2023) 171:156350. doi: 10.1016/J.CYTO.2023.156350 37672863

[31] Mohd Zawawi Z Kalyanasundram J Mohd Zain R Thayan R Basri DF Yap WB. Prospective roles of tumor necrosis factor-alpha (TNF-α) in COVID-19: prognosis, therapeutic and management. Int J Mol Sci. (2023) 24(7):6142. doi: 10.3390/IJMS24076142 37047115 PMC10094668

[32] Saresella M Piancone F Marventano I Hernis A Trabattoni D Invernizzi M. Innate immune responses to three doses of the BNT162b2 mRNA SARS-CoV-2 vaccine. Front Immunol. (2022) 13:947320/FULL. doi: 10.3389/FIMMU.2022.947320/FULL 36072604 PMC9443429

[33] Enssle JC Campe J Moter A Voit I Gessner A Yu W. Cytokine-responsive T- and NK-cells portray SARS-CoV-2 vaccine-responders and infection in multiple myeloma patients. Leukemia. (2024) 38:168. doi: 10.1038/S41375-023-02070-0 38049509 PMC10776400

[34] Cuapio A Boulouis C Filipovic I Wullimann D Kammann T Parrot T. NK cell frequencies, function and correlates to vaccine outcome in BNT162b2 mRNA anti-SARS-CoV-2 vaccinated healthy and immunocompromised individuals. Mol Med. (2022) 28:20. doi: 10.1186/S10020-022-00443-2 35135470 PMC8822735

[35] Escobar A Reyes-López FE Acevedo ML Alonso-Palomares L Valiente-Echeverría F Soto-Rifo R. Evaluation of the immune response induced by coronaVac 28-day schedule vaccination in a healthy population group. Front Immunol. (2021) 12:766278. doi: 10.3389/FIMMU.2021.766278 35173705 PMC8841433

[36] Cunningham L Kimber I Basketter D Simmonds P McSweeney S Tziotzios C. Perforin, COVID-19 and a possible pathogenic auto-inflammatory feedback loop. Scand J Immunol. (2021) 94(5):e13102. doi: 10.1111/SJI.13102 34755902 PMC8646999

[37] Nogimori T Suzuki K Masuta Y Washizaki A Yagoto M Ikeda M. Functional changes in cytotoxic CD8+ T-cell cross-reactivity against the SARS-CoV-2 Omicron variant after mRNA vaccination. Front Immunol. (2023) 13:1081047/FULL. doi: 10.3389/FIMMU.2022.1081047/FULL 36685601 PMC9845949

[38] Kingstad-Bakke B Cleven T Bussan H Yount BL Uraki R Iwatsuki-Horimoto K. Airway surveillance and lung viral control by memory T cells induced by COVID-19 mRNA vaccine. JCI Insight. (2023) 8(22):e172510. doi: 10.1172/JCI.INSIGHT.172510 37796612 PMC10721330

[39] Pérez-Nicado R Massa C Rodríguez-Noda LM Müller A Puga-Gómez R Ricardo-Delgado Y. Comparative Immune Response after Vaccination with SOBERANA® 02 and SOBERANA® plus Heterologous Scheme and Natural Infection in Young Children. Vaccines (Basel). (2023) 11:1636. doi: 10.3390/VACCINES11111636/S1 38005968 PMC10675375

[40] Scarcella M d’Angelo D Ciampa M Tafuri S Avallone L Pavone LM. The key role of lysosomal protease cathepsins in viral infections. Int J Mol Sci. (2022) 23(16):9089. doi: 10.3390/IJMS23169089 36012353 PMC9409221

[41] Majchrzak M Poręba M. The roles of cellular protease interactions in viral infections and programmed cell death: a lesson learned from the SARS-CoV-2 outbreak and COVID-19 pandemic. Pharmacol Rep. (2022) 74:1149. doi: 10.1007/S43440-022-00394-9 35997950 PMC9395814

[42] Zhang S Wang J Wang L Aliyari S Cheng G. SARS-CoV-2 virus NSP14 Impairs NRF2/HMOX1 activation by targeting Sirtuin 1. Cell Mol Immunol. (2022) 19:872–82. doi: 10.1038/s41423-022-00887-w PMC9217730 35732914

[43] Li J Ren JX Liao HP Guo W Feng KY Huang T. Identification of dynamic gene expression profiles during sequential vaccination with ChAdOx1/BNT162b2 using machine learning methods. Front Microbiol. (2023) 14:1138674/FULL. doi: 10.3389/FMICB.2023.1138674/FULL 37007526 PMC10063797

[44] Strasser A Jost PJ Nagata S. The many roles of FAS receptor signaling in the immune system. Immunity. (2009) 30:180. doi: 10.1016/J.IMMUNI.2009.01.001 19239902 PMC2956119

[45] Cummins N Badley A. The TRAIL to viral pathogenesis: the good, the bad and the ugly. Curr Mol Med. (2009) 9:495. doi: 10.2174/156652409788167078 19519406 PMC3149795

[46] Chen W-C Hu S-Y Cheng C-M Shen C-F Chuang H-Y Ker C-R. Evaluating TRAIL and IP-10 alterations in vaccinated pregnant women after COVID-19 diagnosis and their correlation with neutralizing antibodies. Front Immunol. (2024) 15:1415561. doi: 10.3389/FIMMU.2024.1415561 39290698 PMC11405216

[47] Koroleva EP Fu YX Tumanov AV. Lymphotoxin in physiology of lymphoid tissues - implication for antiviral defense. Cytokine. (2018) 101:39. doi: 10.1016/J.CYTO.2016.08.018 27623349 PMC5344785

[48] Mehta G Riva A Ballester MP Uson E Pujadas M Carvalho-Gomes Â. Serological response and breakthrough infection after COVID-19 vaccination in patients with cirrhosis and post-liver transplant. Hepatol Commun. (2023) 7:e0273. doi: 10.1097/HC9.0000000000000273 37870985 PMC10586829

