# Supplementary material for: Beyond interferon gamma - decreased cellular response to COVID-19 vaccination booster in patients with autoimmune inflammatory rheumatic diseases
Source: Front Immunol. 2025 Mar 28;16:1568439. doi: 10.3389/fimmu.2025.1568439 (PMC11986637; doi:10.3389/fimmu.2025.1568439)
Supplement: Supplementary file 1 [file Table1.docx]

Supplementary Material

| **Analyte** | **Test name** | **Manufacturer** | **Detection limit** |
| --- | --- | --- | --- |
| TNF | Human TNF alpha Uncoated ELISA Kit | Invitrogen | 3.9 - 500 pg/mL |
| IL-2 | Human IL-2 ELISA Kit | Proteintech | 15.6 - 1000 pg/mL |
| Perforin | Human Perforin ELISA KIt | ABclonal | 39 - 2500 pg/mL |
| Granzyme B | Human Granzyme ELISA Kit | ABclonal | 15.6 – 1000 pg/mL |

Supplementary Table 1. Details of the kits used for the detection of cytokines involved in cellular response in blood plasma.

| **Gene Symbol** | **Assay ID** | **Gene Name** |
| --- | --- | --- |
| *GAPDH* | Hs02786624_g1 | glyceraldehyde-3-phosphate dehydrogenase |
| *RPS18* | Hs01375212_g1 | ribosomal protein S18 |
| *FAS* | Hs00236330_m1 | Fas cell surface death receptor |
| *FASLG* | Hs00181226_g1 | Fas ligand |
| *CTSC* | Hs00175188_m1 | cathepsin C |
| *CTSH* | Hs00544778_m1 | cathepsin H |
| *NFE2L2* | Hs00975961_g1 | NFE2 like bZIP transcription factor 2 |
| *LTA* | Hs06633590_s1 | lymphotoxin alpha |
| *TNFSF10* | Hs00921974_m1 | TNF superfamily member 10 |
| *CTSB* | Hs00947439_m1 | cathepsin B |
| *GZMA* | Hs00989184_m1 | granzyme A |

Supplementary Table 2. List of analyzed genes.
